# Poikilocytosis and Allied Conditions

**Published:** 1907-05-11

**Authors:** 


### Points in Diagnosis.

### POIKILOCYTOSIS AND ALLIED CONDITIONS.

Poikilocytosis is the term used to denote any condition of the blood in which some of the red corpuscles are no longer round discs, but are of abnormal shape; such ill-shaped red corpuscles are termed poikilocytes, and they are present in the fresh blood as such. They have to be distinguished from artefacts, due to deformation of corpuscles in making, drying, and staining films. The distinction between poikilocytes and artefacts is easy, for the deformity of the latter is either angular from juxtaposition or spiky from crenation, whereas the outline of a poikilocyte is still a smooth curve although the curve is no longer a circle. The outline may be oval, ovoid, pear-shaped, loaf-shaped, and so on, but it is always smooth.

The question arises: What is the diagnostic significance of poikilocytosis? The answer is, that it indicates severe disorder of the blood, but by itself gives no clue whatever to the cause of this disorder. Poikilocytosis is to be expected with any anæmia that is severe. It is seen in its most marked form in the severest anæmia we know, namely, pernicious anæmia; but its presence is no proof that the anæmia in a given patient is of the pernicious type. It is seen in the profound anæmia that directly results from loss of blood, such as post-partum hæmorrhage, extreme epistaxis, gastric or duodenal ulcer; in the anæmia of the late stages of cachexias, such as gastric carcinoma gives rise to; in late stages of spleno-medullary leuchæmia, lymphatic leuchæmia, Hodgkin's disease, splenic anæmia, pseudo-leuchæmia infantum, in the anæmia which results from poisoning by toxic parasites such as ankylostomum duodenale and bothriocephalus latus; in lardaceous disease, infective endocarditis, syphilitic and malarial cachexias, and so on; indeed, in any anæmia which is extreme. If there is any exception to this rule it is chlorosis, for here the reduction in the hæmoglobin may be very profound without poikilocytes appearing in the blood; nevertheless cases of simple chlorosis occur from time to time with well-marked poikilocytosis.

There are two other blood changes which are to be expected when the anæmia, due to whatever cause, is very marked. These are:—

1. Considerable variations in the *size* of the corpuscles. A normal corpuscle is termed a *normocyte*; one which is abnormally small is called a *microcyte*; one which is abnormally large a *megalocyte*. In any of the above diseases, in the later stages when the anæmia has become profound, both microcytes and megalocytes are present in the blood films. By themselves they merely indicate that the anæmia is very great; they are therefore most prevalent in cases of pernicious anæmia, and if, on examining a blood film, the greater number of the corpuscles are found to be meagalocytes, the presumption is that the condition of the patient is one of severe pernicious anæmia. It must always be remembered, however, that megalocytes and microcytes may be found in many other severe anæmias, so that it is not safe to rely on films alone in diagnosing pernicious anæmia. The pathognomonic feature of the latter is the colour index; that is to say, the ratio of the hæmoglobin to the red corpuscles; the red corpuscles must be counted and the hæmoglobin estimated, and the hæmoglobin must be less diminished than are the red corpuscles if the diagnosis of pernicious anæmia is to be made with certainty. Even with pernicious anæmia there need not be many megalocytes and microcytes, any more than there need be poikilocytes; none of these may be a marked feature of the blood film if the condition be discovered early, or if, under treatment, the condition has greatly improved. Megalocytes and microcytes, like poikilocytes, occur chiefly when the anæmia is well marked.

2. The presence of nucleated red corpuscles. In normal blood after birth nucleated red corpuscles are quite absent; their presence always indicates that something is wrong, and badly wrong. The nucleated red cells, if of normal size, are called *normoblasts*; if of small size, *microblasts*; if of large size, *megaloblasts*; if of very large size, especially if more than one nucleus be present, *gigantoblasts*. Gigantoblasts and microblasts are much rarer than normoblasts and megaloblasts, but none of them is pathognomonic of any particular disease. They may be found in all sorts of extreme anæmias, although on the other hand the anæmia may be extreme without nucleated red cells being present. Roughly speaking, the occurrence of nucleated red corpuscles is a sign of worse prognosis than otherwise, but they do not at all necessarily prove that the prognosis is hopeless. Nucleated red cells have been found in the blood after severe hæmorrhage, when the patient was reacting well and was doing her best to reproduce new corpuscles to replace these that had been lost by the hæmorrhage. Recovery in such cases may be complete, and the nucleated red corpuscles may disappear. It has been said that the presence of gigantoblasts means a fatal prognosis, but this is not true. In a case of pseudo-leuchæmia infantum (Von Jeksch's disease) gigantoblasts were present at one time when the child was in extremis, but slow improvement followed, and after the child had been nearly dead for several months it recovered.

Poikilocytes, microcytes, megalocytes, microblasts, normoblasts, megaloblasts, and gigantoblasts are all of them pathological, but none of them is intrinsically characteristic of any particular disease. They may afford some measure of the severity of the anæmia, but they do not indicate its kind. The most that can be said is they are all seen in most marked degree in the latest stages of pernicious anæmia, this being the severest anæmia we know, and that in pernicious anæmia, as a point of distinction between it and other severe anæmias, megalocytes are the preponderating form of abnormal red corpuscles.

